# Multi-omics analysis reveals age-dependent changes in flavor compounds and meat quality in Jiaji ducks between 60 and 70 days of age

**DOI:** 10.1016/j.psj.2025.105796

**Published:** 2025-09-03

**Authors:** Jinyu Qian, Tao Tang, Fengjie Ji, Hanlin Zhou, Lihong Gu, Tieshan Xu, Chengjun Hu

**Affiliations:** a Tropical Crop Genetic Resource Research Institute, Chinese Academy of Tropical Agricultural Sciences, Haikou, Hainan 571101, China; b Institute of Animal Science and Veterinary Medicine, Hainan Academy of Agricultural Sciences, Haikou, China

**Keywords:** Ducks, Flavor compounds, Meat quality, Metabolomics, Transcriptomics

## Abstract

Our previous study demonstrated that duck muscle undergoes rapid development between 60 and 70 days of age. However, the changes and underlying mechanisms governing flavor compound deposition during this period remains unclear. In this study, we compared the meat quality of ducks at 60 days (60D group) and 70 days (70D group) of age. The results showed that the diameter and cross-sectional area of breast muscle fibers were significantly higher, while the cooking loss and drip loss were lower in the 70D group than in the 60D group. Electronic nose analysis revealed a significant difference in muscle alkane content between the two groups. Further analysis indicated that the contents of volatile substances 1-pentanol, nonanal, d-limonene, and 2, 5-dihydroxybenzoic acid in muscle were higher in the 70D group than in the 60D group. Metabolomics identified higher levels of key flavor metabolites such as inosine 5′-monphosphate (IMP), taurine, and anserine in the 70D group. Transcriptomic analysis revealed differentially expressed genes enriched in purine metabolism, taurine and hypotaurine metabolism, and nucleotide metabolism. Integrated analysis of metabolome and transcriptome identified phosphodiesterase 1C, guanylate kinase 1, cysteine dioxygenase type 1, and flavin-containing monooxygenase 3 as regulators of flavor deposition, with phosphoribosyl transferase domain containing 1 specifically linked to IMP. These findings indicate a higher concentration of flavor substances in 70-day-old duck meat, offering a basis for the genetic breeding of high-quality meat ducks breeds.

## Introduction

Meat flavor, a critical determinant of meat quality and consumer preference, is a complex trait influenced by genetic, environmental, and nutritional factors (Prache et al., 2022; Jin et al., 2023). Duck meat is particularly valued for its distinctive flavor profile, which is strongly influenced by slaughter age (Cao et al., 2021; Li et al., 2022a). Volatile flavor compounds are a central determinant of meat quality and consumer acceptance. For instance, the aroma of meat is generated by volatile compounds such as aldehydes (Liu et al., 2023). Although previous studies have characterized flavor-related compounds in mature ducks (Li et al., 2022b), the mechanisms underlying age-dependent deposition of key flavor precursors remain poorly understood. Therefore, elucidating these age-dependent mechanisms is essential for enhancing meat quality in the duck industry.

Flavor deposition in meat is an age-dependent process governed by multiple biochemical pathways (Deng et al., 2022). Age-related changes in duck muscle have been associated with increased deposition of IMP, taurine, anserine, as well as volatile compounds such as nonanal, 1-pentanol, and d-limonene, all of which contribute to umami and fruity aroma notes (Duan et al., 2023; Li et al., 2022a). Moreover, younger ducks typically exhibit higher moisture content and lower intramuscular fat, whereas older ducks accumulate flavor-enhancing metabolites such as unsaturated fatty acids (Duan et al., 2023). Multi-omic association analysis has proven effective in linking genotype to phenotype. For instance, metabolomic studies in ducks have identified hexanal and dimethyl anthranilate as characteristic flavor compounds associated with aging (Duan et al., 2023). Similarly, transcriptomic analysis in chickens has revealed that genes involved in lipid metabolism and energy regulation exhibit age-related expression patterns (Li et al., 2022a; Zhang et al., 2023a). However, whether similar transcriptional networks govern flavor deposition in ducks and how these networks correlate with metabolic shifts during aging remains unclear.

Jiaji duck, an indigenous breed in China, has gained significant consumer preference due to its superior meat quality. Our previous study demonstrated that this breed undergoes rapid muscle development between 60 and 70 days of age (Xu et al., 2025). Additionally, the slaughter age of Jiaji ducks is typically 60 or 70 days. However, the dynamic changes and underlying regulatory mechanisms governing flavor compound deposition during this period remains unclear. Therefore, we investigated the changes of meat quality and deposition of flavor compounds in Jiaji duck muscle. This study provides novel insights into the molecular basis of age-dependent meat quality and offers potential targets for precision genetic selection to enhance duck flavor traits.

## Materials and methods

### Animals and sample collection

A total of 180 one-day-old female Jiaji ducks (Muscovy ducks) were obtained from Hainan Chuanwei Muscovy Duck Breeding Co., Ltd. All ducks were kept under the same conditions with ad libitum access to feed (Table 1) and water. At each sampling time point (60 and 70 days of age), 24 ducks with body weight closest to the group average were randomly selected for sample collection. After breathing anesthesia until unconsciousness, ducks were sacrificed by cutting the carotid arteries. The breast muscles were dissected from the carcass and their weight was recorded. About 5 g breast muscle was immediately snap-frozen in liquid nitrogen or immediately fixed in 4 % paraformaldehyde for further analysis.

**Table 1:** Ingredients and nutrient composition of diets.

Ingredients	Contents (%)	Nutrients	Contents^2^ (%)
Corn	27.50	CP	17.51
Cassava meal	28.00	ME, MJ/kg	11.92
Soybean meal	28.34	CF	3.37
Wheat	8.00	CE	1.99
Wheat bran	3.15	ASH	2.36
Soybean oil	0.70	Ca	0.88
Stone powder	1.20	P	0.53
CaHPO_4_	0.86	Na	0.15
NaCl	0.30	Cl	0.21
Lys	0.10	Lys	0.98
Met	0.35	Met+Cys	0.87
Thr	0.20	Trp	0.40
Trp	0.20	Thr	0.83
Choline chloride	0.10		
Premix^1^	1.00		

1 Provided the following per kg diet: vitamin A, 6000 IU; vitamin D3, 400 IU; vitamin E, 100 IU; vitamin K3, 2.0 mg; thiamin, 5.0 mg; riboflavin, 12 mg; pyridoxine, 5.0 mg; vitamin B12, 0.1 mg; pantothenic acid, 50 mg; niacin, 60 mg; biotin, 0.20 mg; folic acid, 2.5 mg.

2 The contents of crude protein, crude fiber, calcium, and total phosphorus were analyzed.

The experimental design and procedures in this study were reviewed and approved by the Animal Care and Use Committee of the Chinese Academy of Tropical Agricultural Sciences (CATAS-20230802-06).

### Meat quality

Meat quality was estimated by determining muscle shear force, cooking loss, and drip loss. After remove the fascia and fat, the muscle samples were cut parallel to the direction of the muscle fiber with a size of 2.5 cm × 1 cm × 1 cm (length × width × thickness). Shear force was measured using a digital meat tenderness meter (C-LM3B, Tenovo, Beijing, China). For the drip loss analysis, the breast muscle sample was weighed and placed in a container. After cooking for 45 min, the muscle samples were dried and then reweighed. To determine the drip loss of meat, fresh breast muscle samples were weighed (W1), refrigerated at 4 °C, and reweighed (W2) after 48 h. The drip loss of muscle was calculated using the follows formula: (W1 − W2)/W1 × 100 % (Xiong et al., 2021).

### Determination of trace element in muscle

About 100 mg of freeze‐dried sample was digested in 5 mL of acid solution (HNO_3_:HClO_4_ = 4:1) at 180°C for 24 h. After digestion, supernatant liquid was filtered and diluted to a volume of 25 mL with hydrochloric acid and potassium ferricyanide solution (Hu et al., 2021). The trace element including Zn, Fe, Mg, and Se were measured using an inductively coupled plasma optical emission spectrometry (ICP-OES, PRIDE100, Beijing, China).

### Determination of free amino acid in muscle

Muscle free amino acid content was quantified according to previous published methods (Hu et al., 2019). Briefly, approximately 100 mg freeze‐dried muscle samples were hydrolyzed in 10 mL 0.1 mol/L hydrochloric acid solutions for 1 h. The hydrolysate was then centrifuged at 12,000 × *g* for 15 min. Subsequently, 1 mL of the supernatant liquid was diluted with 1 mL water and filtered through a 0.45 μm membrane before analysis. Amino acid analysis was performed using a Hitachi l-8800 amino acid analyzer (Hitachi High-Technologies, Tokyo, Japan)

### Analysis of volatile flavor components in muscle

A gas chromatography mass spectrometry (GC–MS) was used for determination of volatile compounds in duck muscle. Approximately 100 mg freeze‐dried muscle was weighed into a 20 mL headspace vial and sealed immediately with a PTFE/silicone septum cap. Volatile compounds were extracted using a solid-phase microextraction (SPME) fiber (Merck KGaA, Darmstadt, Germany). Prior to extraction, the fiber was conditioned in the GC injection port at 250 °C for 30 min to remove contaminants. For extraction, the SPME fiber was exposed to the sample headspace at 65 °C for 40 min, maintaining a distance of 0.5 cm above the sample. The fiber was then retracted and immediately inserted into the GC inlet for thermal desorption. Analysis was performed on an Agilent 7890B gas chromatograph coupled with a 5977B mass spectrometer (Agilent Technologies, Inc, Santa Clara, CA). The GC was equipped with an HP-5MS capillary column (30 *m* × 0.25 mm × 0.25 μm). The carrier gas was helium at a constant flow rate of 1.0 mL min⁻¹. The oven temperature program was as follows: initial temperature of 30 °C (held for 2 min), increased to 200 °C at 5 °C min⁻¹, then to 220 °C at 2 °C min⁻¹ (held for 10 min). Electron impact ionization was performed at 70 eV, with a quadrupole temperature of 150 °C. Mass spectra were acquired in full-scan mode (m/z 50–550) with a solvent delay of 2 min. Volatile compounds were identified by comparing mass spectra with the NIST 17 library. Semi-quantitative analysis was performed based on peak area normalization.

### Electronic nose (E-nose) analysis

E-nose analysis was performed using a SuperNose-14 (iSenso, New York, USA). Briefly, 2 g of breast muscle were cut parallel to the direction of the muscle fiber with a size of 0.5 cm×0.5 cm×0.5 cm and added into a 20 mL headspace bottle. All the samples were incubated at 80 °C for 15 min before analysis. The self-cleaning time and detection time were 120 s and 60 s, gas flow rate 1.0 L/min. respectively.

### H&E staining

The breast muscle samples were fixed in 4 % paraformaldehyde, paraffin-embedded, and sectioned at 5 μm thickness. The sections were then stained with hematoxylin-eosin (H&E)*.* Microscopic images were captured for each section, with five random fields of view selected per sample. Image J software was used to calculate the diameter and area of muscle fibers.

### Metabolomics analysis

About 50 mg of duck breast muscle tissue was extracted using 0.5 mL of 80 % methanol. After centrifugation at 20,000 g for 15 min, the supernatant was vacuum-dried and redissolved in 100 μL of 80 % methanol. The mixed solution was filtered through a 0.22 m membrane and stored at −80°C before analysis. Metabolomic profiling was conducted using a liquid chromatography-mass spectrometry (LC-MS) system (Agilent 1100, California, USA) according to our previous study (Yang et al., 2024). Metabolites with variable importance in projection (VIP) >1 and *P* < 0.05 were considered as significantly different metabolites (DMs)

### RNA extraction and transcriptome analysis

Total RNA was isolated from breast muscle samples using TRIzol reagent (Sangon Biotech, Shanghai, China). Total RNA was purified with SuperScript II reverse transcriptase (Invitrogen, USA) and reverse-transcribed into cDNA. Sequencing was performed on the illumina NovaseqTM6000 platform (LC-Bio Technolgy, Shanghai, China).

Data analysis was based on previous published methods (Yang et al., 2024). Quality control of sequencing reads was performed using FastQC (v0.23.2), reads with unknown sequences >10 % and quality scores <20 were removed. Gene expression levels were quantified as fragments per kilobase of exon per million mapped reads (FPKM) using StringTie (v2.2.0). Differential gene expression analysis was conducted with DESeq2 (v1.30.0), identifying differentially expressed genes (DEGs) with |log2(fold change)| > 1 and an adjusted p-value < 0.05.

### Integration analysis of transcriptome and metabolome

The two-way Orthogonal Partial Least Squares (O2PLS) analysis was performed using the OmicsPLS package of R (version 4.2.1). The model incorporated all identified differential metabolites and differentially expressed mRNAs to establish cross-omics correlations. Variables with high correlation and weight were preliminarily identified through loading plots, followed by screening of key variables that significantly influenced the other omics dataset. Based on the enrichment analysis results of differential metabolites and differentially expressed genes (DEGs), bar charts were used to display the numbers of differential metabolites and mRNAs in enriched pathways. For mechanistic insights, we mapped these omics features to the KEGG Enzyme database, generating enzyme-centric metabolic networks that explicitly illustrate metabolite-enzyme-gene regulatory relationships.

### Data statistics

Data are represented as mean ± SEM. Statistical analyses were performed using SPSS 20.0 (SPPS Inc., Chicago, IL). The student's *t*-test was performed to analyze differences between the two groups, with *P* < 0.05 was considered as statistically significant.

## Results

### Meat quality and morphological characteristics of breast muscle

**Fig. 1:**
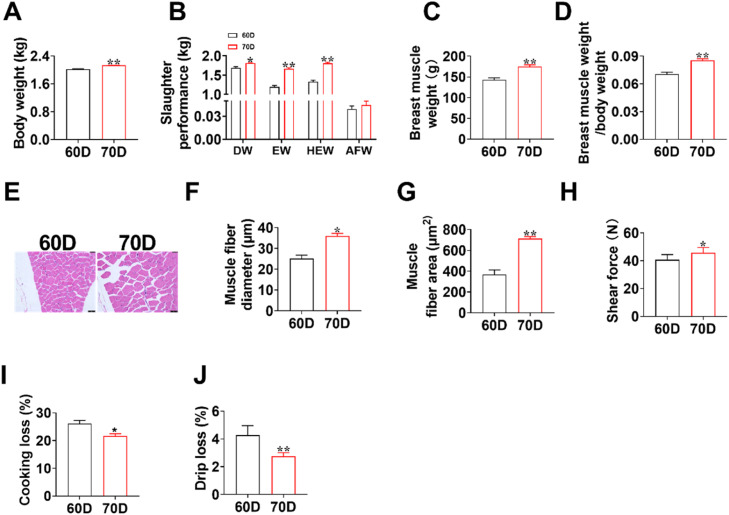
**Comparison of breast muscle fiber morphology and meat quality between 60-day-old and 70-day-old ducks.** (A) Body weight at 60- and 70-day-old. (B) Slaughter performance of ducks. DW: dressed weight; EW: eviscerated weight; HEW: half-eviscerated weight; AFW: abdominal fat weight. (C, D) Breast muscle weight and relative muscle yield. (E-G) Histomorphometry analysis of breast muscle fiber diameter and cross-sectional area. (G-J) Meat quality parameters including shear force, cooking loss, and drip loss. The results are shown as the mean ± SEM. *n* = 24. Independent sample t-tests were performed to determine the significance of differences between groups. *P < 0.05, **P < 0.01.

The results showed that the carcass weight, semi-evisceration, eviscerated, breast muscle weight, and relative breast muscle yield were significantly lower in the 60D group than those in the 70D group (Fig. 1A-D). Moreover, the diameter and cross-sectional area of the breast muscle fibers were significantly lower in the 60D group than in the 70D group (Fig. 1E–G). Compared to the 60D group, the meat shear force was significantly increased in the 70D group (Fig. 1H). However, the cooking loss and drip loss in the 70D group were significantly lower than in the 60D group (Fig. 1I, J).

### Contents of trace element and free amino acid in breast muscle

No significant difference was observed in the content of Fe or Se in breast muscle between the two groups, while the contents of Zn and Mg were lower in the 70D group than in the 60D group (Table 2). Moreover, the contents of Met, Phe, and Trp in breast muscle were significantly higher in the 60D group than in the 70D group, and that of Glu was lower in the 60D group than in the 70D group (Table 3).

**Table 2:** Contents of trace elements in duck breast muscle.

Nutrient levels^1^	60D	70D	*P*-value
Zn (mg/kg)	14.76±0.62^a^	13.31±0.29^b^	0.02
Fe (mg/kg)	46.49±1.47	46.48±0.65	1.00
Mg (mg/kg)	286.75±11.42^a^	256.15±4.68^b^	<0.01
Se (μg/kg)	70.97±4.37	69.85±4.14	0.39

1 Fresh muscle weight basis. Different letter superscripts in the same row indicate significant differences (*P* < 0.05). *n* = 24.

**Table 3:** The free amino acids contents in breast muscle of ducks (μg/g).

Items^1^	60D	70D	*P*-value
CP (%)	20.80±0.21	20.59±0.11	0.34
Asp	97.81±7.07	107.92±4.42	0.21
Glu	88.26±5.33^b^	121.62±6.57^a^	<0.01
Asn	41.87±2.87	49.23±2.96	0.09
Ser	228.7 ± 9.96^a^	175.92±5.17^b^	<0.01
Gln	505.83±45.3	428.14±13.13	0.09
Gly	149.54±5.7	141.87±2.9	0.23
His	101.53±3.6	110.77±4.21	0.14
Tau	528.26±21.21	620.3 ± 53.12	0.10
Arg	460±38.26	520.53±25.28	0.21
Thr	110.07±7.08	113.58±2.66	0.71
Ala	1011.01±23.68	1049.29±26.12	0.31
Pro	80.46±2.28	75.33±2.37	0.14
Tyr	112.94±3.13	106.11±2.64	0.12
Val	116.68±4.92	113.11±3.51	0.62
Met	76.98±2.3^a^	65.8 ± 2.01^b^	<0.01
Ile	99.72±3.72	93.53±2.18	0.28
Leu	143.17±6.22	142.44±5.43	0.94
Phe	117.07±3.51^a^	88.28±4.7^b^	<0.01
Trp	199.34±1.96^a^	50.23±0.79^b^	<0.01
Lys	179.19±11.63	161.84±4.95	0.16
Total AAs	4232.72±87.64	4337.37±134.21	0.50

1 Fresh muscle weight basis. Different letter superscripts in the same row indicate significant differences (*P* < 0.05). *n* = 24.

### Contents of volatile flavor compounds in breast muscle

**Fig. 2:**
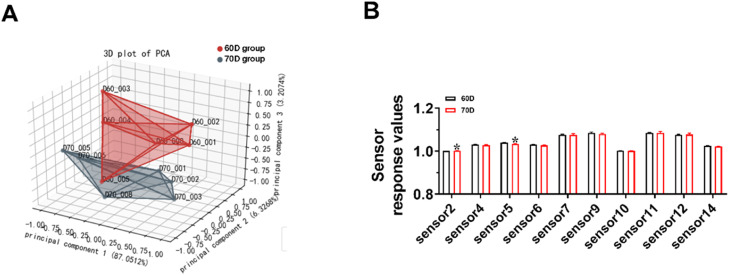
**Electronic nose analysis of meat.** A. PCA analysis between 60D and 70D group. B. The sensor response values of sensor**.** Sensor_2: partial alkane. Sensor_3: Oxygen. Sensor_4: sulfide. Sensor_5: nitrogen (oxygen) compounds, amines. Sensor_6: alcohols, aldehydes, ketones, ethers, etc. Sensor_7: short-chain alkanes. Sensor 9: aromatic compounds, etc. Sensor_10: Hydride. Sensor_11: v0c. Sensor_12: some alkanes and aldehydes. Sensor_14: partial alkanes, olefins. The results are shown as the mean ± SEM. *n* = 24. Independent sample t-tests were performed to determine the significance of differences between groups. *P < 0.05, **P < 0.01.

Electronic nose was employed to measure the contents of volatile compounds related to meat aroma in breast muscle. The results showed that the PCA diagram showed that the two groups of samples were located in different regions (Fig. 2A), indicating a difference in flavor between the two groups. The response values of sensors 2 (sensitive to alkanes) and sensor 5 (sensitive to nitrogen oxide) were higher in the 60D group than that in the 70D group (Fig. 2B).

GC-MS was used to analysis the differences of volatile substances between the two groups. The results showed that the relative contents of 1-pentanol, nonylaldehyde, d-limonene, and 2, 5-dihydroxybenzoic acid in duck breast muscle were significantly higher in the 70D group than in the 60D group (Table 4).

**Table 4:** Relative content of volatile substances in breast muscle of ducks.

Items	RT (min)	60D	70D	*P*-value
1-Pentanol	3.9	1.00±0.04^b^	1.39±0.10^a^	<0.01
Hexanal	4.4	1.00±0.08	1.20±0.16	0.22
2,3-Butanediol	4.8	1.00±0.06	0.98±0.07	0.80
1-Hexanol	5.9	1.00±0.09	0.93±0.13	0.68
2-Heptanone	6.4	1.00±0.04	0.92±0.07	0.31
Heptanal	6.7	1.00±0.04	1.00±0.07	0.98
2,3-Octanedione	9.1	1.00±0.08	0.77±0.12	0.10
2-Octenal	11.5	1.00±0.06	0.87±0.11	0.27
Nonylaldehyde	13.2	1.00±0.10^b^	1.68±0.29^a^	0.02
Decanal	16.7	1.00±0.06	0.99±0.00	0.90
Silanediol	3.0	1.00±0.18	1.03±0.10	0.91
D-Limonene	10.6	1.00±0.05^b^	3.11±0.23^a^	<0.01
Hexamethylenimine	11.7	1.00±0.16	0.92±0.04	0.78
1,4-Octadiene	11.9	1.00±0.08	1.01±0.03	0.96
Dodecane	19.9	1.00±0.07	0.81±0.18	0.26
Homopiperazine	12.9	1.00±0.13	0.84±0.09	0.39
Thiourea	2.1	1.00±0.02	0.98±0.01	0.60
Cycloheptane	8.7	1.00±0.09	1.03±0.07	0.86
Furan	9.4	1.00±0.06	1.01±0.10	0.90
2,5-Dihydroxybenzoic acid	31.2	1.00±0.05^b^	1.32±0.04^a^	<0.01
Cyclopropane	12	1.00±0.07	1.16±0.11	0.23
Oxalic acid	23.1	1.00±0.16	0.57±0.10	0.19
Cyclotetrasiloxane	9.6	1.00±0.04	1.05±0.07	0.48

Different letter superscripts in the same row indicate significant differences (*P* < 0.05). *n* = 24.

### Metabolomics analysis

**Fig. 3:**
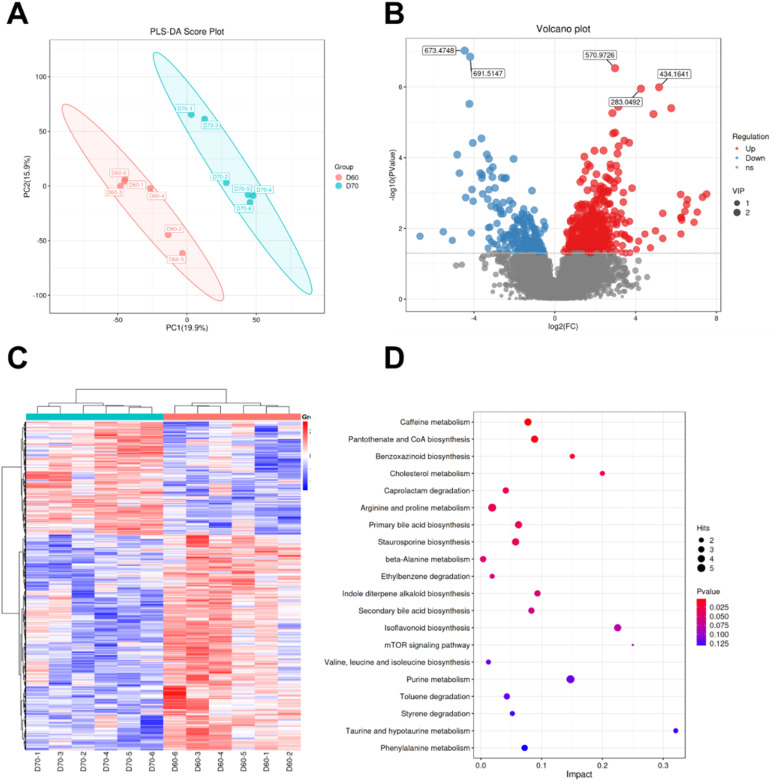
**Metabolomic profiling of duck breast muscle between 60-day (D60) and 70-day (D70) groups.** (A) Partial least squares-discriminant analysis (PLS-DA) score plot demonstrating group separation. Volcanic maps (B) and heat maps (C) illustrate the differences in metabolites between the two groups. (D) KEGG pathway enrichment analysis of differential metabolites (top 20 enriched pathways were shown).

To further investigate the effects of age on meat flavor compounds, the changes of metabolite in the breast muscle were analyzed. The results showed that there is a significant difference in metabolite profile between the two groups (Fig. 3A). Compared to the 60D group, a total of 438 DMs were significantly up-regulated and 231 DMs were significantly down-regulated (Fig. 3B, C). KEGG enrichment analysis showed that DMs were enriched in arginine and proline metabolism, primary bile acid biosynthesis, beta-Alanine metabolism, cholesterol metabolism, pantothenate and CoA biosynthesis, and staurosporine biosynthesis pathways (Fig. 3D). Flavor compounds including IMP, anserine, taurine, citraconic anhydride, 2-furoic acid, 7-methylxanthosine, panaquinquecol 1, and 1‑hydroxy-2-naphthoic acid were significantly higher in the 70D group than in the 60D group (Table 5).

**Table 5:** Relative content of metabolites related to meat flavor in breast muscle of ducks.

Items	60D	70D	*P*-value
7-Methylxanthosine	1.00±0.22^b^	2.52±0.48^a^	0.02
Panaquinquecol 1	1.00±0.37^b^	3.85±1.03^a^	0.03
Triacetic acid	1.00±0.16^b^	4.90±1.64^a^	0.02
6-Pentyl-2H-pyran-2-one	1.00±0.05	1.26±0.10	0.05
Hydrocinnamic acid	1.00±0.05^b^	1.37±0.10^a^	0.01
Sinapyl alcohol	1.00±0.17^b^	1.72±0.19^a^	0.02
Vanilloyl glucose	1.00±0.10^b^	1.59±0.16^a^	0.01
Tyramine	1.00±0.09^b^	1.35±0.11^a^	0.03
5-Hydroxyconiferaldehyde	1.00±0.05^b^	1.32±0.11^a^	0.02
Uracil	1.00±0.04^b^	5.61±0.31^a^	0.04
Taurine	1.00±0.07^b^	2.10±0.46^a^	0.04
4-Tert-Butylcatechol	1.00±0.05^b^	1.36±0.10^a^	0.01
Indole-3-carboxilicacid-O-sulphate	1.00±0.16^b^	2.22±0.34^a^	0.01
2-Furoic acid	1.00±0.23^b^	2.79±0.56^a^	0.01
Citraconic anhydride	1.00±0.36^b^	2.68±0.45^a^	0.02
2-Furoic acid	1.00±0.23^b^	2.79±0.56^a^	0.01
1-Hydroxy-2-naphthoic acid	1.00±0.12^b^	1.90±0.19^a^	<0.01
Anserine	1.00±0.06^b^	1.30±0.07^a^	0.01
IMP	1.00±0.14^b^	1.59±0.23^a^	0.05

Different letter superscripts in the same row indicate significant differences (*P* < 0.05).

### Transcriptome analysis

**Fig. 4:**
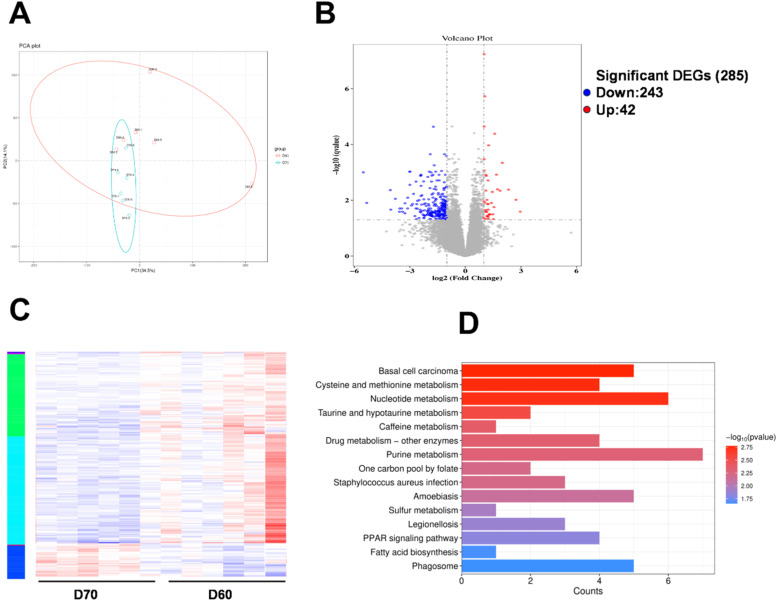
**Transcriptomic analysis of duck breast muscle.** (A) Principal components analysis of genes between two groups. Volcanic maps (B) and Heat maps (C) illustrate the DEGs between the two groups. (D) KEGG enrichment pathway of DEGs.

The PCA revealed distinct clustering patterns between the two groups (Fig. 4A). We found 285 DEGs between 60D group and 70D group, of which 42 genes were up-regulated and 243 genes were down-regulated (Fig. 4B). The top 100 DEGs in the breast muscle were shown in the heat map (Fig. 4C). Pathway enrichment analysis revealed DEGs were enriched in purine metabolism, nucleotide metabolism, cysteine and methionine metabolism, and taurine and hypotaurine metabolism (Fig. 4D).

### Transcriptome and metabolome integration analysis

**Fig. 5:**
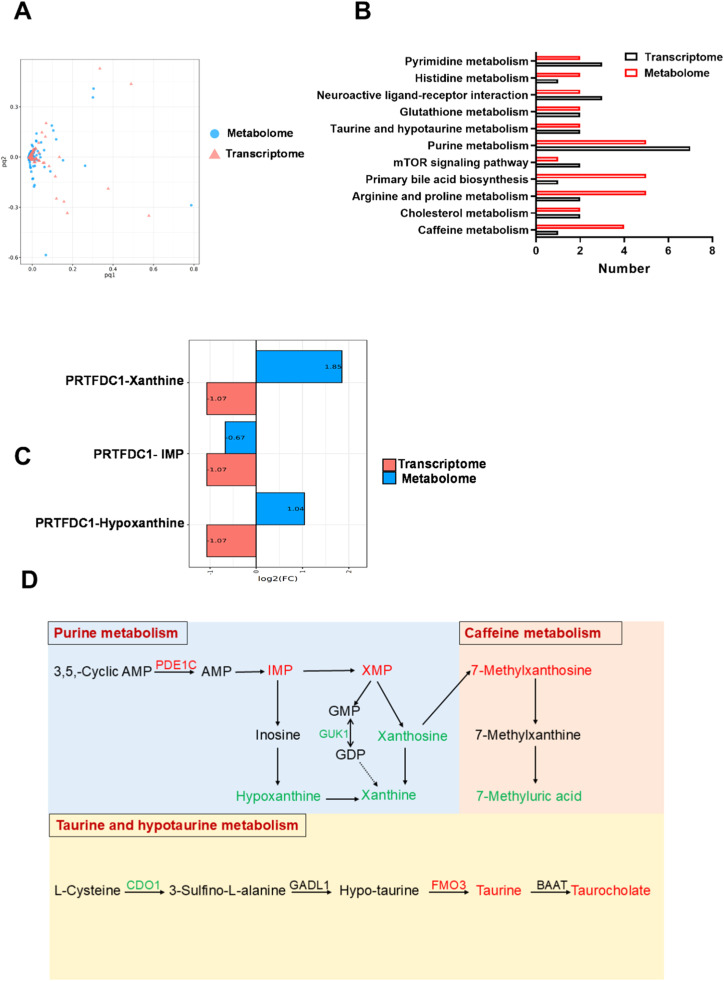
**Correlation analysis of transcriptomics and metabolomics.** A. O2PLS score plots representing the relationship between metabolomic (blue) and transcriptomic (red) datasets. The circle and triangle showed the name of DMs and DEGs, respectively. The red line represents positive correlation, and the green line represents negative correlation. B. The number of differential metabolites and differential mRNAs in the enriched pathways. C. The enzyme-substrate correlation analysis. The x-axis represents the log_2_(FC) values in different omics studies, while the y-axis represents the correspondence between mRNA and metabolism in enzymatic reactions. Red indicates the transcriptome, and blue indicates the metabolome. The length of each bar reflects the magnitude of differential expression (or abundance) in the respective omics dataset, indicating a greater impact on the enzymatic reaction. D. Analysis of key metabolites and genes that regulate meat quality and flavor formation of duck meat. Metabolites and genes marked in red indicate upregulation, while those marked in green indicate downregulation.

We conducted an integrated transcriptomics and metabolomics analysis to reveal the mechanisms of flavor compound deposition in breast muscle. The two-way orthogonal PLS (O2PLS) analysis revealed significant correlations between the gene and metabolite expression within each group (Fig. 5A). KEGG analysis of DMs and DEGs showed that there were 11 co-enriched KEGG pathways, namely purine metabolism, caffeine metabolism, taurine and hypotaurine metabolism, glutathione metabolism, cholesterol metabolism, arginine and proline metabolism, primary bile acid biosynthesis, mTOR signaling pathway, histidine metabolism, pyrimidine metabolism, and neuroactive ligand-receptor interaction (Fig. 5B). The enzyme substrate correlation analysis revealed that the phosphoribosyl transferase domain containing 1 (PRTFDC1) contributes to IMP deposition (Fig. 5C). Integration analysis of metabolome and transcriptome revealed the potentially functional genes influencing flavor compositions and metabolic pathways including phosphodiesterase 1C (PDE1C), guanylate kinase 1 (GUK1), cysteine dioxygenase type 1(CDO1), and flavin-containing monooxygenase 3 (FMO3) (Fig. 5D).

## Discussion

Duck meat, a significant source of high-quality protein in the human diet, is primarily chosen by consumers based on its quality and taste. Meat quality and flavor of duck meat is influenced by a multitude of factors, including genetics, nutrition, age, and gender (Zhang et al., 2024). Our previous study demonstrated that duck muscle undergoes rapid development between 60 and 70 days of age (Xu et al., 2025). Subsequently, we systematically evaluated the meat quality parameters during this critical developmental period. The results revealed that the body weight, dressed weight, eviscerated weight, breast muscle weight, breast muscle fiber diameter, and cross-sectional area were significantly higher in the 70D group than in the 60D group. Aligning with our prior observation, a significant age-dependent increase was observed in both the mass and myofiber cross-sectional area of breast muscles in ducks (Xu et al., 2025). Moreover, the meat shear force was significantly increased in the 70D group relative to the 60D group, suggesting the meat tenderness was significantly decreased in the 70D group. This is consistent with previous study reporting that meat tenderness was significantly decreased as the age of chicken increased from 65 d to 75 d (Deng et al., 2022). Enhanced muscle water content contributes to improved tenderness, juiciness, firmness, and overall appearance of the meat (Kasperek et al., 2023; Li et al., 2022a). Water-holding capacity, which reflects fresh meat’s ability to retain moisture after slaughter, is inversely correlated with drip and cooking losses (Barbut, 2024; Li et al., 2022a). In this study, the drip and cooking losses of the breast muscle in the 60D group were significantly higher than those in the 70D group, indicating superior water retention and tenderness in the duck meat of the 70D group. To further elucidate the impact of age on meat quality, we analyzed the contents of trace element in breast muscle and found that the contents of Zn and Mg were lower in the 70D group than in the 60D group. The results of the present study are consistent with the findings of Cao et al. (2021), who reported that Zn and Mg contents in duck meat decreased with age. Zn is intricately linked to the synthesis and bioactivity of growth hormone and insulin-like growth factor (IGF) (Hamza et al., 2012). The anabolic promotion of muscle growth by these hormones might accelerated the metabolic utilization of Zn. Mg is an essential activator for key enzymes in glycolysis and the tricarboxylic acid cycle (Abdollahi et al., 2022). It is crucially utilized to catalyze energy metabolism processes, which support muscle cell proliferation and hypertrophy. These results suggested that increased utilization of trace elements during muscle rapid development. Collectively, the results indicate that the meat quality of 70-day-old ducks is superior to that of 60-day-old ducks.

Amino acids are instrumental in determining the quality and flavor of meat (Kelly and Pearce, 2020). Glu, a crucial flavor-enhancing amino acid, plays an indispensable role in meat flavor (Mukherjee et al., 2023). In this study, the free Glu content in the 70D group was markedly higher than in the 60D group, suggesting that the breast muscle in the 70D group possessed superior flavor quality compared to the 60D group. Beyond influencing meat flavor, free amino acids also serve as the fundamental building blocks of protein (Amorim et al., 2025). Here, we found that the muscle in 70D group had significantly lower levels of free Ser, Met, Phe, and Trp than in the 60D group. The concentration of free amino acids in muscle is determined by the balance between protein synthesis and degradation (Ishihara et al., 2021). The reduction in these free amino acids in breast muscle indicated an enhanced incorporation into muscle proteins during rapid growth.

The volatile flavor is one main part of meat flavor and plays a crucial factor in determining meat quality (Jin et al., 2023). Therefore, electronic nose was employed to measure the presence of volatile compounds related to the meat aroma in breast muscle. The results showed that the two groups showed difference in alkanes. Further analysis by GC-MS indicated that the meat from 70D group exhibited significantly higher concentrations of 1-pentanol, nonylaldehyde, d-limonene, and 2,5-Dihydroxybenzoic acid. 1-pentanol, known for its sweet aroma and low odor threshold, significantly contributes to the overall food flavor (Li et al., 2022a). Nonylaldehyde is a key odorant contributing to the citrus-like aroma, and d-limonene is the major volatile compound responsible for the characteristic aroma of orange peel in meat (Sheng et al., 2025). In line with our study, previous study showed that meat total aldehydes contents in old ducks were significantly higher than those in young ducks (Duan et al., 2023). These results indicate that 1-pentanol, nonylaldehyde, d-limonene, and 2,5-dihydroxybenzoic acid contributes to the characteristic flavor of ducks from different ages. To further reveal the differences of flavor compounds between the two groups, we collected breast muscle from the two groups for nontargeted metabolomic analysis. The results showed that flavor compounds including IMP, anserine, taurine, citraconic anhydride, 2-furoic acid, 7-methylxanthosine, panaquinquecol 1, and 1‑hydroxy-2-naphthoic acid were significantly higher in 70D group than those in 60D group. Previous research reporting IMP in muscle presented an upward trend with age in chicken (Li et al., 2022a). IMP, a key nucleotide associated with umami taste, contributes to the savory flavor profile of duck meat (Kang et al., 2025). Anserine and taurine are important compounds in muscle with antioxidant properties that ensure the stability of the oxidation of proteins and lipids in muscle (Liu et al., 2024; Zhao et al., 2024). 7-Methylxanthosine, a purine derivative, could modify nucleotide turnover. The increased 7-methylxanthosine in muscle suggests modifications in purine metabolism pathways, potentially influencing meat tenderness through nucleotide-mediated proteolysis. These findings demonstrate an age-dependent enhancement in the deposition of flavor compounds in duck meat.

The accumulation of flavor compound is a result of dynamic physiological and metabolic transformations in muscle tissue, which mediated by complex gene regulatory networks (Huang et al., 2025). Therefore, transcriptomic analysis was employed to reveal the intricate mechanisms governing flavor compounds deposition in muscle. KEGG enrichment analysis of DEGs showed that the significantly enriched pathways were mostly related to purine metabolism, nucleotide metabolism, cysteine and methionine metabolism, and taurine and hypotaurine metabolism. By comparing the KEGG pathways of DMs and DEGs, it was found that there were 11 common pathways, mainly involved in purine, caffeine, taurine and hypotaurine, amino acids and fatty acids metabolism, suggesting these compounds may be important factors affecting the breast muscle quality and flavor. Subsequently, we focused on these differential metabolites enriched into the most significant path ways and identified the key target genes regulating flavor compounds. Compared to the 60D group, the expression level of *PDE1C* was significantly up-regulated, and that of *GUK1* in 70D group were significantly down-regulated in the breast muscle. These two genes were involved in purine metabolism. PDE1C is a subfamily of PDE super enzyme families that can hydrolyze cAMP (Zhang et al., 2023b). An increase in PDE1C expression may lead to elevated local AMP levels, thereby enhancing the supply of IMP precursor molecules. In taurine and hypotaurine metabolism, the expression level of *FMO3* was significantly up-regulated, and that of *CDO1* in 70D group were significantly down-regulated in the breast muscle. CDO1 downregulation in 70D muscle may reflect feedback inhibition by taurine accumulation. Furthermore, reduced CDO1 activity could shunt cysteine toward glutathione synthesis, thereby indirectly stabilizing taurine pools (Liu et al., 2024). These results suggested that the changes of expression of *PDE1C, GUK1, CDO1*, and *FMO3* might contributed the IMP and taurine accumulation in breast muscle. Furthermore, the enzyme-substrate correlation analysis revealed that the *PRTFDC1* contributes to IMP deposition. PRTFDC1, a hypoxanthine-guanine phosphoribosyltransferase (HPRT) homolog, specifically interacted with the hypoxanthine and guanine, thus converting hypoxanthine and guanine into their corresponding nucleoside monophosphate (Welin et al., 2010). These results suggested that PRTFDC1 may indirectly affect IMP deposition by binding to substrates or interacting with other HPRT enzymes. Nevertheless, future studies are required to investigate the intricate regulatory networks.

## Conclusions

The results have demonstrated that the contents of flavor substances and the meat quality in 70-day-old Jiaji ducks are superior to those in 60-day-old ducks. Flavor compounds IMP, taurine, anserine, 7-methylxanthosine, triacetic acid, 5-hydroxyconiferaldehyde, 1-pentanol, nonanal, d-limonene, and 2, 5-dihydroxybenzoic acid presented an upward trend with age. The enzyme-substrate correlation analysis revealed that the *PRTFDC1* contributes to IMP deposition. These findings provide molecular insights into the regulatory mechanisms of age-related flavor compounds deposition in duck meat. Future studies could investigate the role of *PRTFDC1* in IMP deposition.

## CRediT authorship contribution statement

**Jinyu Qian:** Writing – review & editing, Writing – original draft, Visualization, Software, Investigation, Formal analysis, Data curation. **Tao Tang:** Writing – review & editing, Software, Methodology, Investigation, Data curation. **Fengjie Ji:** Software, Methodology, Investigation, Data curation. **Hanlin Zhou:** Methodology, Formal analysis, Data curation. **Lihong Gu:** Writing – review & editing, Methodology, Investigation, Data curation, Conceptualization. **Tieshan Xu:** Writing – review & editing, Validation, Project administration, Methodology, Investigation, Formal analysis. **Chengjun Hu:** Writing – review & editing, Writing – original draft, Project administration, Methodology, Investigation, Funding acquisition, Conceptualization.

## Disclosures

The authors declare that they have no known competing financial interests or personal relationships that could have appeared to influence the work reported in this paper.

## Data Availability

Data will be made available on request.
